# Injury of flexor halluics longus tendon in amateur marathon runners results in abnormal plantar pressure distribution: observational study

**DOI:** 10.1186/s12891-024-07169-8

**Published:** 2024-01-20

**Authors:** Jianan Chen, Ping Zhang, liting Hou, Hailun Bao, Junfei Li, Jian Zhao

**Affiliations:** 1 Department of Radiology, The Third Hospital of Hebei Medical University, Shijiazhuang, China; 2 Hebei Province Biomechanical Key Laboratory of Orthopedics, The Third Hospital of Hebei Medical University, Shijiazhuang, China

**Keywords:** Plantar pressure, Marathon runners, Flexor halluics longus tendon injures, Biomechanics

## Abstract

**Objective:**

To analyze the changes of plantar pressure in amateur marathon runners with flexor halluics longus (FHL) tendon injury using the Medtrack-Gait plantar pressure measurement system, and to explore whether the plantar pressure data can be used as an index for the diagnosis of injury.

**Methods:**

A total of 39 healthy amateur marathon runners without any ankle joint symptoms were recruited. Dynamic and static plantar pressure data were measured using the pressure plate of Medtrack-Gait. According to MRI imaging findings, whether the FHL tendon was injured or not was judged, and the dynamic and static data were divided into the injury group and control group. The data with statistically significant differences between the two groups were used to make the receiver operating characteristic (ROC) curve.

**Result:**

The maximum contact area (PA) of the first metatarsal(M1) region, the maximum load-bearing peak value (PW) and the time pressure integral (PMPTI) of the second metatarsal(M2) region in the injury group were lower than those in the control group, respectively (*P* < 0.05). The maximum contact area (PA) of the fifth metatarsal(M5) region was higher than that in the control group (*P* < 0.05). The area under curve (AUC) value of the ROC curve of the PA of M1 region, the PW and PMPTI of M2 region were statistically (*P* < 0.05).

**Conclusion:**

FHL tendon injury resulted in decreased PA in M1, decreased PW and PMPTI in M2, and increased PA in the M5 region, suggesting that FHL tendon injury resulted in a force shift from the medial to the lateral side of the foot. The PA of M1, PW and PMPTI of M2 have certain diagnostic value for early FHL injury in amateur marathon runners.

## Background

Marathons have become popular among runners, with about several million people participating in marathons each year, most of whom are amateur marathon runners, and the number is rising year by year [1, 2]. Because it is a long-distance running sport that challenges the limits of endurance, injuries to the musculoskeletal system of the lower limbs are very common. It has been proved that among the running-related injuries (RRIs), knee, ankle-foot and calf have the highest percentage of injuries [3, 4]. The ankle joint is one of the major joints in lower limb movement and is susceptible to injuries to bone, cartilage, tendons and ligaments during exercise due to a variety of internal and external factors [2]. The flexor hallucis longus (FHL) tendon, which crosses the back of the medial ankle to the sole of the foot, is essential in helping people walk and move. Sports injuries to the FHL tendon are most likely to occur in classical ballet dancers [5–10]. Some scholars have named this injury “dancer’s tendinitis” [9]. In addition to ballet dancers, cases of FHL injuries are more commonly reported in long distance runners [5–8, 10–13]. The injury may be caused by excessive plantarflexion of the ankle joint due to repetitive push-off maneuvers during prolonged running [2, 7, 10, 14], which is inevitable in marathon training. Therefore, early diagnosis of FHL injuries in amateur marathon runners is particularly important. Magnetic resonance imaging (MRI) should be an important tool to aid in the diagnosis of foot and ankle diseases and helps to assess the FHL tendon status [7, 8, 10]. However, due to the mild symptoms or asymptomatic of some amateur marathon runners, MRI could not be performed in time, and there is a high incidence of peritendinous effusion in asymptomatic patients [2, 10].

Gait analysis research based on plantar pressure have become a major research priority in the field of biomechanics. Gait analysis studies have become a tool for evaluation, not only for kinesiology or basic biomechanical studies, but also for diagnosis, monitoring functional recovery and musculoskeletal rehabilitation [15]. By analyzing the distribution of various values of plantar pressure changes during various types of sports, it is possible to predict and diagnose certain diseases, such as diabetic foot, knee osteoarthritis [15–17]. The 39 amateur marathon runners were recruited for this study and grouped according to whether or not the FHL tendon was injured on MRI images. The aim was to investigate whether the alteration of plantar pressure distribution in amateur marathon runners could be used as a sensitivity indicator to evaluate the occurrence of disease and provide a reference value for the early diagnosis of RRI in amateur marathon runners.

## Materials and methods

### General information

39 amateur marathoners were recruited as the subjects of this study by random recruitment. They all met the inclusion and exclusion criteria. The 39 eligible volunteers consisted of 29 males and 10 females. Their ages ranged from 29 to 50 years, with a mean ± standard deviations of 40.8 ± 5.6 years; Body mass indices (BMIs) ranged from 17.3 to 28.6 kg/m^2^, with a mean of 22.7 ± 3.0 kg/m^2^; Running age is mean of 3.9 ± 2.0 years; The distance of running each month is mean of 274.3 ± 102.2 km. All volunteers underwent routine ankle MRI examination, plantar dynamic and static measurements with gait analyzer. Then the dynamic and static plantar data were grouped according to whether the FHL tendon was injured by imaging examination. Dynamic data were included according to single-foot FHL tendon injury, and the data that could not judge the status of FHL tendon due to image quality were excluded. Static data were included according to bipedal FHL tendon injury. The data of single foot FHL tendon injury or the FHL tendon status could not be determined due to image quality were excluded. Both groups of FHL tendons without injury data were used as the control group. All volunteers were highly compliant, informed and voluntarily signed the informed consent form for this study.

### Inclusion and exclusion criteria of amateur marathon runners

Inclusion criteria: [1] Age 25–50 years; [2] Completed at least 1 time the regular full marathon or 2 half marathons; [3] Not to participate in the competition as a specially invited athlete or domestic registered athlete; [4] Long-term regular running, running times ≥ 3 times a week, each running distance ≥ 10 km, or monthly running ≥ 100 km; [5] No training or strenuous exercise within 1 week before the examination.

Exclusion criteria: [1] Lower limb muscle and ankle injuries during the examination; [2] Presence of diseases involving the muscular system, such as diabetes, muscular dystrophy, etc. [3]. Both lower limb muscles and ankle joints have a history of trauma and surgical operations; [4] Have contraindications to MRI.

### Gait data measurement

**Fig. 1:**
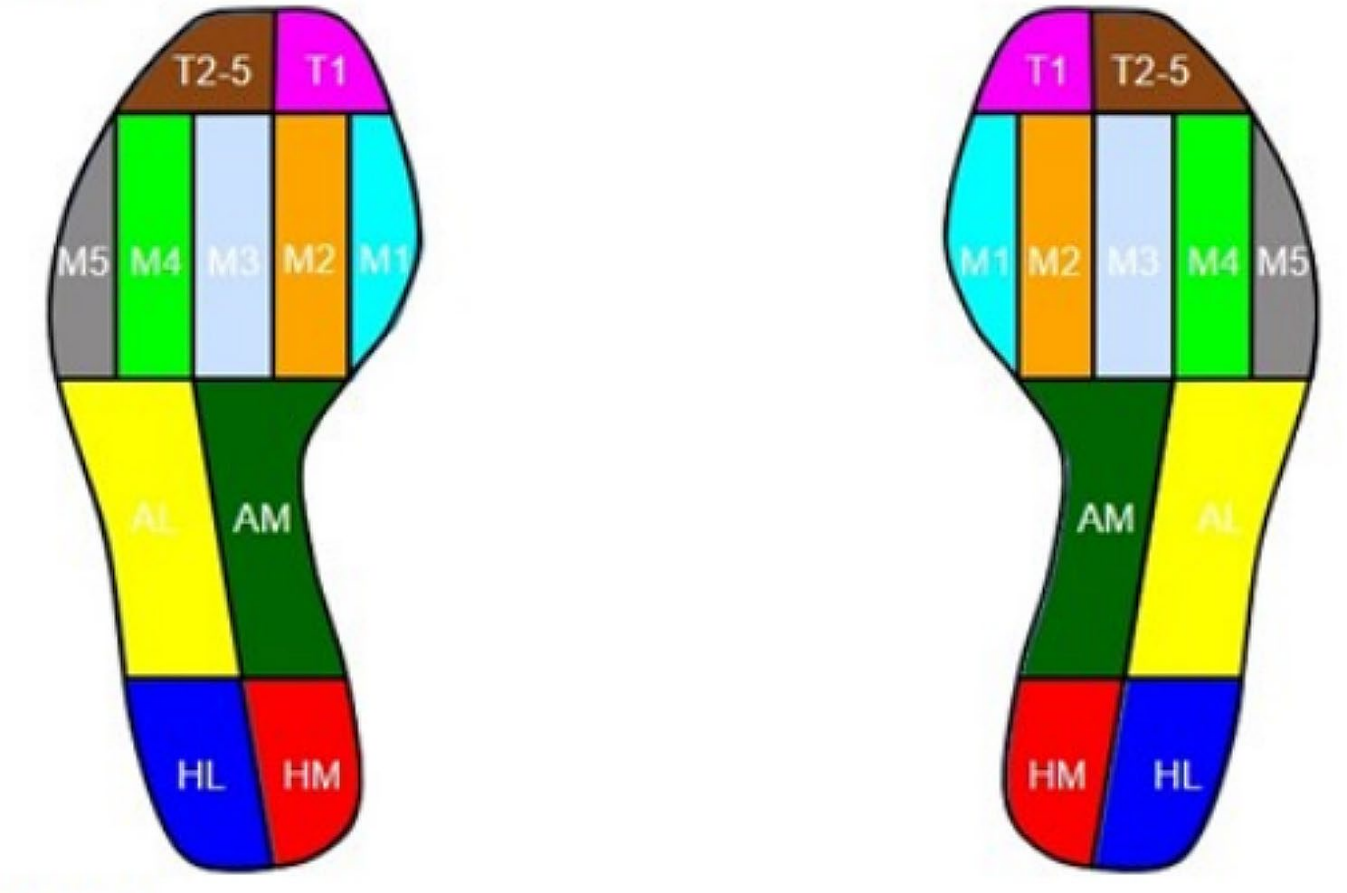
The plantar partition

The Medtrack-Gait (Xinkang Biological, China) plantar pressure system was used to measure the plantar pressure on the 1.2 m pressure plate (EVA runway, piezoresistive sensor array, acquisition frequency 400fps). Eleven anatomical zones were automatically identified by the Medtrack-Gait software. These areas were defined as medial heel (HM), lateral heel (HL), medial arch of the foot (AM), lateral arch of the foot (AL), metatarsal 1–5 (M1, M2, M3, M4 and M5), the hallux (T1) and toe 2–5 (T2–5) (Fig. 1). The volunteers began to measure the data while standing barefoot in their natural state on the device inside the static measuring circle of the platen. The first 15s allowed the volunteers to maintain a stable standing position on the pressure plate. Then select more stable data within 15-30 s (maximum measurement time is 30s) and save the data. Then, the volunteers were instructed to walk on the pressure plate with their personal habits, walking back and forth three times at a uniform speed in a straight line to measure the dynamic data. The volunteers were all acclimated before the test. Three groups of complete dynamic data onto the left and right feet were taken, and the left and right foot data were selected once for comparative analysis. Both dynamic and static plantar data were automatically generated by the device’s system. The system comes with analysis software to analyze the collected data files and obtain the corresponding raw data.

### MRI scanning method and sequence parameters

All the volunteers underwent MRI on a 3.0-T scanner (SIGNA Architect, GE Healthcare, American) with a 16-channel phased-array ankle coil. Scanning protocols were as follows: [1] Sagittal fat-suppressed proton density weighted imaging (FS-PDWI) TR, 2324.0 ms; TE, 42.9 ms; FOV, 150.0 mm × 150.0 mm; flip angle, 111°; and slice thickness, 3.0 mm; [2] Coronal fat-suppressed proton density weighted imaging (FS‐PDWI) TR, 2462.0 ms; TE, 50.05 ms; FOV, 140.0 mm × 140.0 mm; flip angle, 111°; and slice thickness, 3.0 mm; [3] Axial fat-suppressed proton density weighted imaging (FS‐PDWI) TR, 2326.0 ms; TE, 44.4 ms; FOV, 140.0 mm × 140.0 mm; flip angle, 111°; and slice thickness, 4.0 mm; [4] T1-weighted imaging (T1WI) sequence in axial plane TR, 845.0 ms; TE, 9.05 ms; FOV, 140.0 mm × 140.0 mm; flip angle, 111°; and slice thickness, 4.0 mm.

### Image analysis

**Fig. 2:**
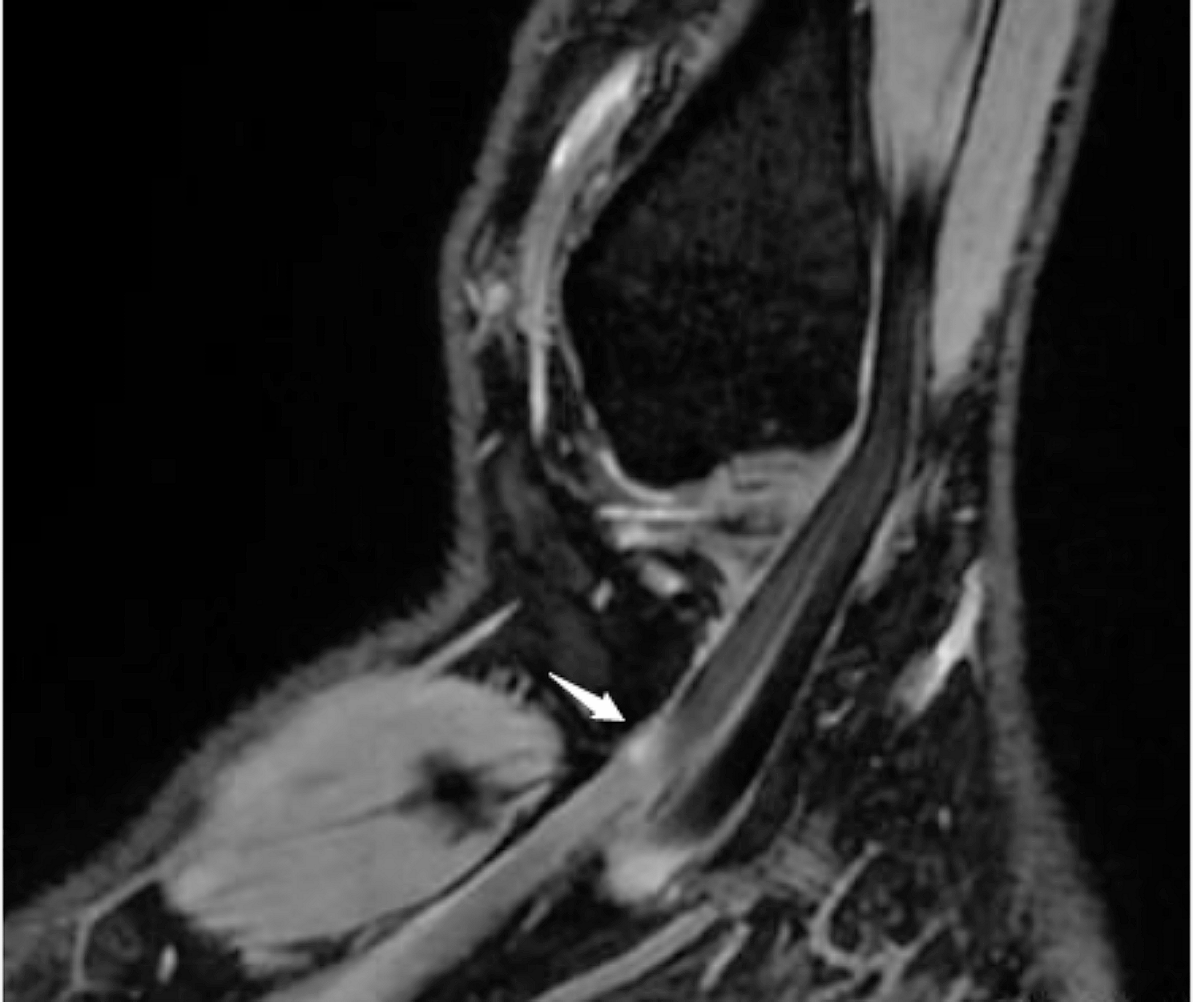
This image shows an MRI of a volunteer with a flexor halluics longus (FHL) tendon injury(Increased signal in the tendon indicated by the arrow, suggesting tendon injury)

The images were analyzed independently by two diagnostic radiologists who each had 5 or more years of experience in diagnostic musculoskeletal imaging. The criteria for the diagnosis of FHL tendon injury are as follows: (1) the T2 signal within the tendon is increased(Fig. 2), which can be observed on the images at all locations; (2) Whether the peripheral soft group is accompanied by edema as an indirect sign. Most of the amateur marathon volunteers had asymptomatic FHL tendon injuries, and they also had no accidental injuries recently to their lower limbs or ankles. Therefore, the tendon injuries found in FHL were all chronic injuries. In case of discrepancies between the radiologists’ reports concerning the findings, the final diagnosis result was determined by the third senior professional musculoskeletal radiologist.

### Statistical analysis

Statistical analyses were conducted using SPSS 25.0 statistical software. General data such as height, weight, and age were compared between the two groups of static plantar data using independent samples t-test. Comparison of dynamic and static plantar data groupings in the FHL tendon injury group and the control group: Shapiro-Wilk test was used for the normality test, and the independent sample t-test was used for data that conformed to normal distribution; The mann - Whitney U test was used for data that did not conform to normal distribution. We recorded the mean difference and 95% confidence interval (95%CI). The difference median and 95%CI were calculated using Hodges-Lehmann. Receiver Operating Characteristic (ROC) curves were constructed for parameters with statistically significant differences. To calculate the sensitivity, specificity, area under curve (AUC) and 95% CI to evaluate the accuracy of diagnosing FHL tendon injury in amateur marathon runners. Analysis used an α of 0.05; *P* values < 0.05 were considered statistically significant.

## Results

A total of 73 dynamic plantar pressures on the feet were included from 39 amateur marathon runners, and the rest were excluded because the image quality could not be determined whether they were injured or not. According to the imaging findings are divided into FHL tendon injury group and the control group. The PW, PMPTI values in the M2 area and the PA values in the M1 area of the injury group were significantly lower than those of the control group (*p* < 0.05), and the PA values of M5 area were significantly greater than those of the control group (*p* < 0.05). The rest of the data were not statistically different (*p* > 0.05) (Tables 1, 2, 3 and 4). According to the inclusion and exclusion criteria of static plantar data, 9 volunteers were included in the static FHL tendon injury group, 18 volunteers were included in the control group, and the rest were excluded. There was no statistically significant difference between the general information of the static data injury group and the control group (*P* > 0.05) (Table 5). The comparison of all data of static plantar was not statistically significant (all *P* > 0.05) (Table 6).

**Fig. 3:**
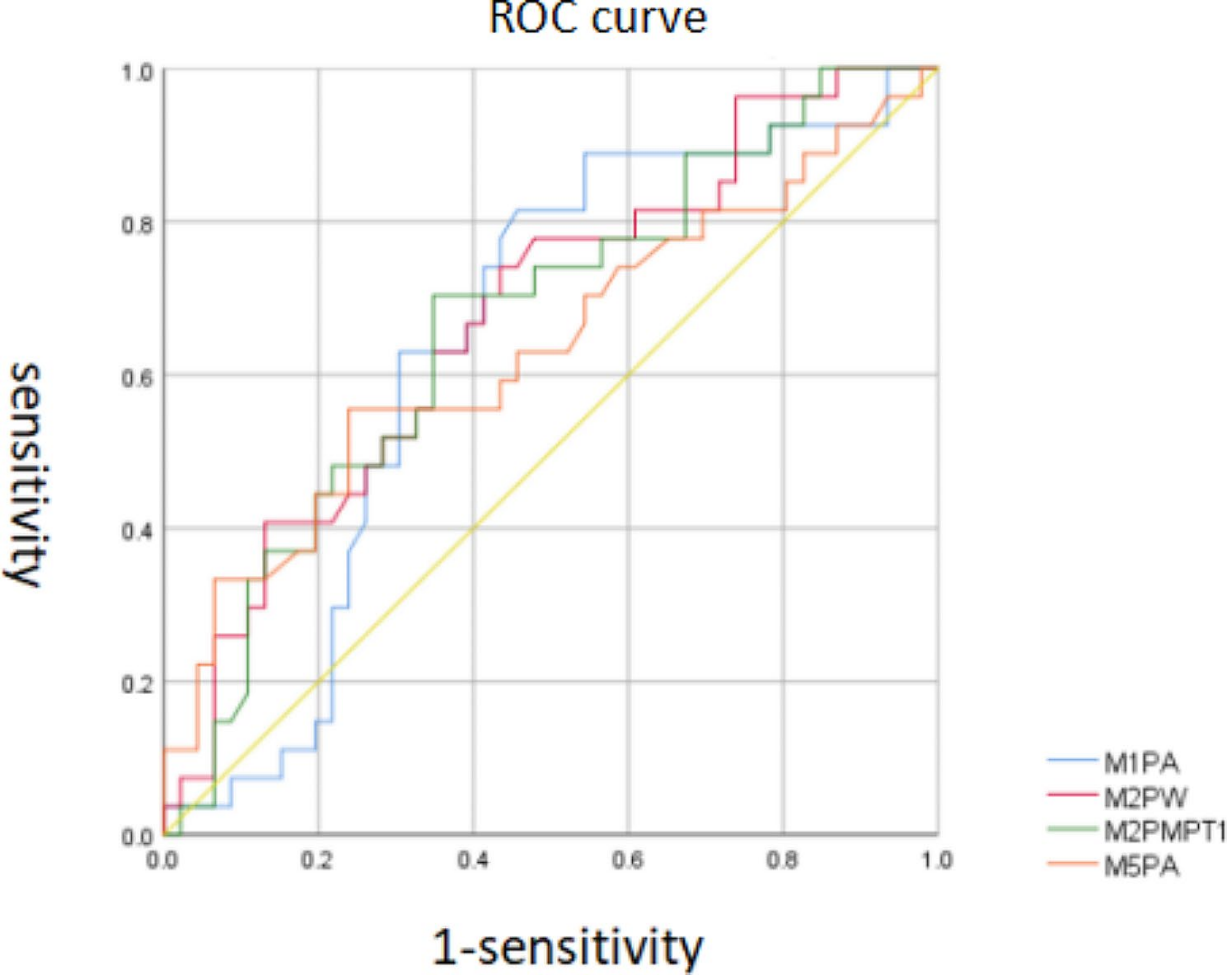
ROC curve

The area of ROC curve showed that the three groups of data were above the reference line and away from the reference line (Fig. 3). The statistical results showed that AUC of PW and PMPT values in M2 area and PA values in M1 area was statistically significant (*p* < 0.05) (Fig. 2)(Table 7). The maximum Youden index was 0.306 at a cut-off value of 15.48 for the PW value in the M2 zone, which corresponds to a sensitivity of 74% and a specificity of 57%. It means that the sensitivity of diagnosing FHL tendon injury is 74% and the specificity is 57% when the PA value of dynamic plantar pressure in M2 area is less than 15.48 kg. The maximum Youden index was 0.356 at a cut-off value of 12 for PMPTI in M2, which corresponds to a sensitivity of 70% and a specificity of 65%. It means that when the PMPTI value of dynamic data M2 is less than 12Mpa*s, the sensitivity of diagnosing FHL tendon injury is 70% and the specificity is 65%. The maximum Youden index was 0.358 at a cut-off value of 12.92 for the PA value in the M1 zone, which corresponds to a sensitivity of 81% and a specificity of 54%. It means that the sensitivity of diagnosing FHL tendon injury is 81% and the specificity is 54% when the PA value of dynamic plantar pressure in M1 area is less than 12.92cm^2^.

**Table 1:** PW: the maximum load-bearing peak

Partition		PW(kg)	Difference and 95%CI	Z or t	*P*-value
FA	I	55.21 ± 9.85	−1.49(−6.57 to 3.59)	t = 0.586	0.56
	C	56.7 ± 10.86			
TA	I	7.01(4.86,10.83)	−0.4(−2.03 to2.25)	Z = 0.04	0.968
	C	8.6(5.02,10.94)			
MA	I	44.54 ± 7.41	−2.39(−6.52 to 1.75)	t = 1.15	0.254
	C	46.93 ± 9.16			
CA	I	8.19(2.91,10.15)	−0.67(−3.64 to 1.54)	Z = 0.617	0.537
	C	5.87(2.5,9.83)			
HA	I	36.28 ± 7.06	0.60(−3.16 to 4.36)	t = 0.317	0.752
	C	35.68 ± 8.17			
T1	I	7.57(4.46,9.79)	0.22(−1.41 to 2.25)	Z = 0.326	0.745
	C	6.59(4.66,9.26)			
T2−5	I	0.87(0.25,2.61)	−0.13(−0.7 to 0.27)	Z = 0.589	0.556
	C	0.83(0.5,1.5)			
M1	I	6.66 ± 3.4	1.15(−2.95 to 0.64)	t = 1.28	0.203
	C	7.81 ± 3.87			
M2	I	13.73 ± 2.82	−2.73(−3.64 to−0.57)	t = 2.73	**0.008**
	C	15.84 ± 3.36			
M3	I	12.72(11.31,14.51)	0.45(−1.38 to 2.45)	Z = 0.291	0.771
	C	12.88(10.46,16.53)			
M4	I	9.62(6.38,13.01)	−0.74(−2.73 to 1.01)	Z = 0.828	0.407
	C	8.44(6.63,11.45)			
M5	I	3.02 ± 1.58	0.42(−0.21 to 1.04)	t = 1.31	0.192
	C	2.6 ± 1.12			
CM	I	0(0,0.01)	0(0 to 0)	Z = 0.858	0.391
	C	0(0,0.02)			
CL	I	8.19(2.91,10.14)	−0.69(−3.71 to 1.39)	Z = 0.628	0.53
	C	5.87(2.4925,9.83)			
HM	I	17.19 ± 3.74	0.4(−1.5 to 2.3)	t = 0.42	0.676
	C	16.79 ± 4.04			
HL	I	19.14 ± 4.59	0.26(−1.39 to 1.91)	t = 0.311	0.987
	C	19.17 ± 5.33			

Dynamic plantar pressure data comparison, normal distribution data use mean ± SD, non-normal distribution data use M(P_25,_P_75_). I:Injure group; C: Control group.

**Table 2:** PMP: peak pressure

Partition		PMP(Kpa)	Difference and 95%CI	Z or t	*P*-value
FA	I	324.5(270.84,327.71)	8.41(−22.97 to 42.61)	Z = 0.606	0.545
	C	327.53(301.65,379.83)			
TA	I	163.76(114.26,198.28)	−0.32(−29.14 to 33.59)	Z = 0.754	0.982
	C	157.68(111.88,218.33)			
MA	I	278.71(245.41,326.31)	3.1(−27.41 to 33.59)	Z = 0.194	0.846
	C	304.6(249.14,338.7)			
CA	I	100.78 ± 56.4	20.68(−1.05 to 42.42)	t=−1.898	0.062
	C	80.1 ± 36.75			
HA	I	234.78(207.53,321.33)	11.13(−20.35 to 43.3)	Z = 0.788	0.43
	C	250.21(220.69,304.94)			
T1	I	136.76(114.26,198.28)	−0.385(−29.91 to 35.59)	Z = 0.326	0.973
	C	157.68(111.87,218.33)			
T2−5	I	37.49(24.73,75.53)	2.925(−10.49 to 16.51)	Z = 0.491	0.623
	C	44.24(33.15,72.28)			
M1	I	159.44 ± 68.83	−10.08(−38.17 to 18.02)	t = 0.715	0.477
	C	169.52 ± 50.9			
M2	I	253.38(231.01,284.56)	20.95(−7.34 to 49.55)	Z = 1.394	0.163
	C	280.11(237.07,324.32)			
M3	I	246.35(221.27,301.4)	21.67(−9.83 to 50.46)	Z = 1.28	0.201
	C	276.32(225.11,328.04)			
M4	I	184.37(154.68,251.04)	−21.61(−57.35 to9.18)	Z = 1.371	0.17
	C	171.75(139.03,218.06)			
M5	I	128.88 ± 54.53	3.57(−23.37 to 30.51)	t = 0.264	0.793
	C	125.31 ± 56.41			
CM	I	2.77(0,8.22)	0(−0.37 to 2.58)	Z = 0.012	0.99
	C	2.61(0,11.67)			
CL	I	100.78 ± 56.4	22.55(1.02 to 44.08)	t = 2.089	0.069
	C	78.23 ± 35.91			
HM	I	234.78(207.53,285.04)	−0.915(−29.95 to 28.45)	Z = 0.069	0.945
	C	239.72(198.06,298.38)			
HL	I	232.0(299.06,290.93)	5.64(−24.46 to 36.17)	Z = 0.371	0.71
	C	239.1(202.12,296.56)			

Dynamic plantar pressure data comparison, normal distribution data use mean ± SD, non-normal distribution data use M(P_25,_P_75_). I:Injure group; C: Control group.

**Table 3:** PA: maximum contact area of the plantar

Partition		PA(cm^2^)	Difference and 95%CI	Z or t	*P*-value
FA	I	108.78 ± 18.81	−0.73(−9.44 to 7.97)	t = 0.618	0.867
	C	109.52 ± 17.54			
TA	I	22.46 ± 5.64	1.0(−1.47 to 3.47)	t = 0.809	0.42
	C	21.45 ± 4.78			
MA	I	56.29(50.23,58.86)	0.89(−2.42 to 3.83)	Z = 0.508	0.611
	C	56.25(52.2,59.83)			
CA	I	23.15(15.53,25.73)	−0.35(−3.56 to 3.49)	Z = 0.166	0.868
	C	21.06(15.92,26.18)			
HA	I	43.25 ± 5.35	0.93(1.78 to 3.64)	t = 0.685	0.496
	C	42.32 ± 5.75			
T1	I	15.89 ± 2.68	−0.27(−1.69 to 1.14)	t = 0.386	0.701
	C	16.16 ± 3.053			
T2−5	I	6.79 ± 3.68	1.04(−0.42 to 2.49)	t = 1.418	0.204
	C	5.75 ± 2.55			
M1	I	11.88(9.98,12.86)	1.41(0.06 to 2.6)	Z = 2.046	**0.041**
	C	13.05(11.07,14.95)			
M2	I	13.6(12.01,14.73)	0.43(−0.43 to 1.32)	Z = 1.12	0.263
	C	14.05(12.82,15.11)			
M3	I	11.8 ± 1.19	−0.19(−0.9 to 0.51)	t = 0.54	0.591
	C	12 ± 1.6			
M4	I	11.5 ± 1.52	0.02(−0.69 to 0.74)	t = 0.068	0.946
	C	11.48 ± 1.44			
M5	I	7.68 ± 1.85	0.87(0.11 to 1.62)	t = 2.282	**0.026**
	C	6.82 ± 1.37			
CM	I	0.03(0,,0.18)	0(0 to 0.9)	Z = 0.53	0.596
	C	0.06(0,0.41)			
CL	I	23.15(15.53,25.73)	−0.48(−3.76 to 3.16)	Z = 0.274	0.784
	C	20.91(15.92,25.91)			
HM	I	20.12 ± 2.79	0.13(−1.30 to 1.55)	t = 0.18	0.858
	C	19.99 ± 3.034			
HL	I	23.1 ± 3.21	0.26(−1.369 to 1.91)	t = 0.311	0.757
	C	22.84 ± 3.53			

Dynamic plantar pressure data comparison, normal distribution data use mean ± SD, non-normal distribution data use M(P_25,_P_75_). I:Injure group; C: Control group.

**Table 4:** PMPTI: time pressure integral

Partition		PMPTI(Map*s)	Difference and 95%CI	Z or t	*P*-value
FA	I	13.52(12.21,16.3)	0.47(−0.85 to 1.95)	Z = 0.754	0.451
	C	13.85(12.26,17.19)			
TA	I	2.76(1.54,4.14)	0.01(−0.89 to 0.85)	Z = 0.046	0.964
	C	2.71(1.74,5.41)			
MA	I	10.85 ± 2.88	−0.86(−2.54 to 0.82)	t = 1.021	0.311
	C	11.71 ± 3.77			
CA	I	2.83(1.07,4.8)	−0.47(−1.35 to 0.46)	Z = 0.914	0.361
	C	1.875(0.1,3.05)			
HA	I	6.5(3.67,8.44)	0.62(−0.89 to2.27)	Z = 0.754	0.451
	C	6.55(4.24,9.87)			
T1	I	2.24(1.74,4.55)	0.17(−0.78 to 1.09)	Z = 0.371	0.71
	C	2.74(1.65,4.31)			
T2−5	I	0.53(0.17,1.45)	0.02(−0.39 to 0.22)	Z = 0.16	0.873
	C	0.58(0.28,1)			
M1	I	5.1(2.44,8.01)	0.785(−0.8 to 2.32)	Z = 1.063	0.288
	C	6.1(3.79,7.96)			
M2	I	9.86(8.63,14.13)	2.39(0.41 to 4.83)	Z = 2.36	**0.018**
	C	13.12(9.66,17.42)			
M3	I	12.51(9.82,15.65)	0.61(−1.75 to 3.0)	Z = 0.463	0.644
	C	12.34(9.92,17.93)			
M4	I	10.58 ± 5.29	0.91(−1.40 to 3.23)	t = 0.786	0.435
	C	9.67 ± 4.48			
M5	I	4.17 ± 2.43	0.39(−0.64 to 1.43)	t = 0.756	0.452
	C	3.78 ± 1.96			
CM	I	0(0,0.05)	0(0 to 0)	Z = 0.269	0.788
	C	0(0,0.07)			
CL	I	2.78 ± 2.04	0.4(−0.6 to 1.38)	t = 0.788	0.433
	C	2.38 ± 2.05			
HM	I	6.19 ± 2.78	0.78(−2.39 to 0.93)	t = 0.967	0.337
	C	5.97 ± 3.6			
HL	I	5.99(3.55,8.15)	0.63 (−0.88 to 2.27)	Z = 0.846	0.398
	C	6.16(3.89,9.29)			

Dynamic plantar pressure data comparison, normal distribution data use mean ± SD, non-normal distribution data use M(P_25,_P_75_). I:Injure group; C: Control group.

**Table 5:** General data comparison of the static grouping

Information	Injury group (mean ± SD)	control group (mean ± SD)	Mean differences and 95%CI	t	*p*-value
Age(year)	38.89 ± 4.46	42.06 ± 2.26	3.17(−1.67 to7.999)	1.350	0.189
Weight(kg)	67 ± 12.68	71.17 ± 9.76	4.17(−4.9 to 13.23)	0.947	0.353
BMI(kg/m2)	22.5 ± 3.46	23.68 ± 2.48	1.18(−1.19 to 3.57)	1.033	0.312
Running age(year)	4 ± 2.65	4.19 ± 1.9	0.19(−1.63 to 2.02)	0.220	0.828
Monthly running distance(km)	334.44 ± 148.5	243.98 ± 87.26	−90.56(−183.56 to 2.44)	2.005	0.056

**Table 6:** Static plantar data comparison

Static plantar data	Injury group M(P25,P75)	Control group M(P25,P75)	Z	*p*-value
STTL(cm)	93.12(60.56,152.19)	92.72(74.44,155.11)	0.257	0.797
XTL(cm)	67.02(43.16,95.18)	54.69(48.39,79.6)	0.720	0.471
YTL(cm)	51.22(33.11,87.63)	66.69(48.72,101.06)	1.080	0.280
XMSD(cm)	0.03(0.02,0.06)	0.04(0.03,0.06)	0.472	0.637
YMSD(cm)	0.03(0.02,0.04)	0.04(0.03,0.07)	1.929	0.054
EA(cm)	2.4(1.18,4.66)	2.58(1.83,3.4)	0.643	0.520
SA(cm^2^)	1.98(1.24,2.33)	2(1.53,2.86)	1.183	0.237
EL(°)	0.53(0.49,0.75)	0.65(0.42,0.82)	0.232	0.817
XAWCD(cm)	1.81(1.37,2.66)	2.04(1.47,2.51)	0.309	0.758
YAWCD(cm)	3.72(2.21,4.24)	2.77(1.55,4.7)	0.694	0.487
SS(m/s)	0.04(0.02,0.06)	0.04(0.03,0.05)	0.695	0.487
XSS(m/s)	0.03(0.02,0.04)	0.03(0.02,0.03)	0.154	0.877
YSS(m/s)	0.02(0.01,0.03)	0.03(0.02,0.05)	1.621	0.105
TLPUA(cm/cm^2^)	41.62(17.91,109.97)	34.77(30.96,50.66)	0.154	0.877

STTL: Shake total trajectory length; XTL: X trajectory length; YTL: Y trajectory length; XMSD: x maximum shake diameter; YMSD: x maximum shake diameter; EA: Exposed area; SA: Shake angle; EL: ellipsoid; XAWCD: X axis weight center deviation; YAWCD: Y axis weight center deviation; SS: Shake speed; XSS: X shake speed; YSS: Y shake speed; TLPUA: Trajectory length per unit area;

**Table 7:** AUC of ROC curve and 95% CI

Partition	AUC[95%CI]	P	Cut-off	Youden index	Sensitivity(%)	Specificity(%)
M1PA	0.644[0.514,0.774]	0.041	12.92cm^2^	0.358	81.5	54.3
M2PW	0.676[0.549,0.802]	0.013	15.48 kg	0.306	74.1	56.5
M2PMPTI	0.666[0.583,0.794]	0.018	12Map*s	0.356	70.4	65.2
M5PA	0.635[0.494,0.774]	0.056	7.61cm^2^	0.317	55.6	76.1

## Discussion

The aim of this study was to apply quantitative plantar pressure values for early diagnosis of lower limb injuries in amateur marathon runners. The plantar pressure can be used as a pioneer index for clinical diagnosis or imaging examination when the injury is asymptomatic. Gait analysis is a branch of biomechanics. Through development, gait analysis has gradually changed from qualitative research to quantitative research on kinematics and dynamics of human movement. Plantar pressure is the one of the most important focuses of quantitative studies on gait analysis dynamics. Mild tendon injuries caused by long-term long-distance running in amateur marathon runners are usually in a subclinical state and are often ignored. Furthermore, continuing repetitive movements can lead to irreversible damage. In this study, amateur marathon runners had no obvious clinical symptoms, but there were many FHL tendon injuries actually, which was also similar to previous studies [2]. Other studies have shown that lower extremity biomechanical changes increase the risk of sports injury [18]. This all makes quantitative plantar pressure data clinically feasible for the early diagnosis of lower extremity injuries in amateur marathon runners.

The results of our study found that among amateur marathon runners with FHL tendon injury, compared with those without injury, the PW value and PMPTI value in M2 area and the PA value in M1 area of dynamic plantar pressure data decreased, and the PA value in M5 area increased. Therefore, we speculate that the function and anatomy of FHL may be related to changes in plantar pressure. The FHL functions primarily in active plantarflexion at the first metatarsophalangeal and hallux interphalangeal joints [7, 10]. Active flexion hallux during movement resists the dorsal curvature of the forefoot [7, 19], stabilizes the longitudinal arch, maintains toe-ground contact during heel lift, and reduces forces under the metatarsal head [9, 19]. Romash [13] in his study has shown that in gait, the metatarsophalangeal joint of the great toe hallux is dorsiflexed just before push-off. The FHL muscle helps propel the limb forward, and the tendon undergoes tension during dorsiflexion of the metatarsophalangeal joint. Combined tension and compression of the tendon may compromise the blood supply to the tendon. Such prolonged repeated stress, without sufficient healing or recovery time, may lead to injury. Moreover, the same is true in the marathon. Repeated plantarflexion of the ankle joint leads to overuse of the FHL tendon. Moreover, due to the long-term training of marathon runners, the overused tendon does not recover well, which increases the risk of tendon injury significantly [2, 7]. In addition, a study have shown that the FHL tendon is more likely to be damaged by running posture with a forefoot landing and running age [2]. When the overflexion of the ankle plantar reaches a certain load, athletes experience discomfort during the push-off phase of the gait cycle, while the athlete may report discomfort in the first metatarsophalangeal joint when the FHL is injured and in a subclinical state [14]. Because there are no obvious symptoms of FHL injury, athletes may make minor adjustments in the landing mode of the plantar region involved in FHL tendon when they feel unwell, in order to compensate for the pressure load in the area where the FHL tendon acts during running. Thus the characteristics of the kinematic changes also lead to the use of non-optimal mechanics of the lower extremity in running. These changes alter the lower extremity loading and thus the plantar pressure in the injury group.

Ferris, Sharkey [20] et al. used a static cadaver model and an experimental device of independent development that can simulate the physiological muscle forces in the cadaver foot while recording ground reaction forces and plantar pressure patterns. This study can control the role of each flexor muscle in the movement and investigate the pressure distribution during heel rise. The results showed that plantar flexion without FHL caused statistically significant reduction in the contact area and force of the great toe, with a corresponding increase in the contact area and force under the forefoot metatarsal region. This is different from the reduced PW value in the M2 region and PA value in the M1 region of FHL injury as a result of our study. We concluded that all the FHL injuries in our study were in the state of asymptomatic and mild injury, while the FHL could function normally. The plantar flexion of the foot and ankle is involved by a variety of muscles, and all of them have a compensatory role in the plantar flexion. Ferris, Sharkey [20] also indicated that forefoot and toe contact area, peak and mean pressures, and peak pressures in the forefoot metatarsal region were significantly correlated with the second metatarsal plantar. Suggesting that pressure loading in the M2 region is the result of a mixture of factors. Whereas there was no difference in PW and PA values between the whole foot (FA) and the total metatarsal regions (MA) in our study. The PA value of M5 increases, and its area increases, the greater the range of force action. Thus, the load-bearing force of the M2 region and the contact area of the M1 region was reduced, releasing the pressure in the anatomical location of the injured FHL tendon. This may partially explain the transfer of the reduction in contact area and force from the medial foot to the lateral foot caused by subclinical FHL tendon injury in this study. Thereby the area of the force in the M5 region has increased, but the specific mechanism is still unclear. The PMPTI value is the pressure-time integral, which is the accumulation of force per unit area in time. In this study, the PW value of M2 area in the FHL injury group decreased, and other data were not significantly different, so the PMPTI value also decreased accordingly.

Some scholars have studied the plantar pressure of amateur marathon runners in static conditions [21]. Their study measured plantar angle, plantar axis, and forefoot and rearfoot plantar pressure of the dominant and non-dominant extremity in the static condition. The results showed that only the forefoot pressure data of the sole of the dominant limb were different, while the rest of the static plantar data were not significantly different, which was similar to the results of our study. Our results showed no difference in the static data between the two groups, but we believe that this is related to the weaker FHL tendon function in the static condition [22]. A systematic review study addressed the relationship between static plantar posture and plantar pressure [23]. It shows that foot posture can also cause changes in plantar pressure. Variation in static foot posture is a risk factor for lower limb injury. The feet with abnormal foot posture, such as pes planus (low medial longitudinal arch) or pes cavus (high medial longitudinal arch), are associated with increased odds of lower limb injury. But inconsistencies in the foot posture classification, gait analysis protocols and plantar pressure analysis methods they included compromised the ability to draw definitive conclusions from the data. So they are certain limitations.

Previously, there have been some studies on the plantar pressure changes in marathon runners. Nagel, Fernholz [24] used a capacitive platform to measure plantar pressure changes in athletes while walking barefoot before and after marathon running. The aim was to investigate the effects of exhausting long-distance running on foot-loading characteristics during barefoot walking. Their study focused on pressure in the metatarsal region. It concluded that plantar pressure in the metatarsal region increases after long runs, which may be associated with stress-induced fatigue fractures of the metatarsals in long distance runners. Nagel’s study is similar to ours in that a pressure plate was applied to measure plantar pressure data barefoot, rather than using a sensor insole inside a running shoe. Although sensor insoles are more widely used and may be more beneficial to directly study the kinetic parameters in shoes during running. But their data will be affected by some other external factors. In addition, the pressure insole in the shoe may change the running posture of the foot, and the athlete may feel discomfort during running. Although barefoot measurement can only indirectly reflect long-term effects, it is not affected by external factors and can measure the most realistic data on the bottom of the foot. Nevertheless, it has its shortcomings, such as its measurement area is usually small, and only a few steps or a single step of pressure parameters can be measured at a time. Hohmann, Reaburn [25] applied sensing insoles to a study similar to Nagel’s. Interestingly, their results differed from Nagel’s in that they showed no significant difference in plantar pressure between amateur marathon runners before and after running. Willems, De Ridder [26] also calculated the medio-lateral pressure distribution ratio at different phases of the roll-off. They indicated an altered plantar pressure pattern after a long-distance run. Several of these parameters have been identified as risk factors for running injuries as stress fractures, PFPS, exercise-related lower leg pain and ankle sprains. All these studies were conducted to investigate the relationship between plantar pressure and lower extremity sports injuries in long-distance runners. Nevertheless, few studies like ours have applied quantitative data on plantar pressure dynamics to the diagnosis of injuries in athletes. It would be fascinating to recognize the differences between plantar pressures in different regions of the foot and to understand whether foot pressures vary in amateur marathoners and whether various factors may have an effect on these pressures. The data obtained may help to understand the injuries associated with distance running and may also help to improve the performance of these athletes. The application of quantitative data of plantar pressure to the early diagnosis of sports injury in marathon runners has great application prospect. Future research should build a more extensive database to make the plantar pressure data more complete for various injuries so that such related injuries can be diagnosed and treated at an early phase.

### Limitation

There are also some limitations to this study: the sample size included was relatively small; The dynamic plantar pressure was single foot data, and the influence of weight, age, gender and other factors could not be eliminated. Considering the cross-sectional nature of this study, we are not able to accurately measure the FHL damage threshold. They will be improved as much as possible in the following study.

## Conclusion

In conclusion, dynamic plantar pressures of amateur marathon runners with asymptomatic FHL tendon injury were different compared with control. The maximum load-bearing peak and time pressure integral in the second metatarsal region and the maximum plantar contact area in the first metatarsal region of dynamic plantar pressure data decreased. The maximum plantar contact area in the fifth metatarsal region was increased. It was possible that the injury caused a transfer of force from the medial to the lateral side of the foot. Moreover, the maximum load-bearing peak, time pressure integral of the second metatarsal region and the maximum plantar contact area in the first metatarsal region can be used as a diagnostic indicator of early FHL tendon injury in amateur marathon runners. Plantar pressure may be served as an alternative methods for early detection of flexor hallucis longus tendon injury.

## Data Availability

The datasets used and analyzed during the current study are available from the corresponding author on reasonable request.

## Abbreviations

95% CI: 95% confidence interval
AL: Lateral arch of the foot
AM: Medial arch of the foot
AUC: The area under curve
BMI: Body mass indices
C: Control group
FHL: Flexor halluics longus
HL: Lateral heel
HM: Medial heel
I: Injure group
M1, M2, M3, M4 and M5: Metatarsal 1–5
MRI: Magnetic resonance imaging
PA: Maximum contact area of the plantar
PMP: Peak pressure
PMPTI: Time pressure integral
PW: The maximum load-bearing peak
ROC: Receiver operating characteristic
RRIs: Running-related injuries
T1: The hallux
T2–5: Toe 2–5
